# Cardiac MRI for the assessment of early chemotherapeutic cardiac injury and toxicity

**DOI:** 10.1186/1532-429X-17-S1-P370

**Published:** 2015-02-03

**Authors:** Bina Franklin, Veronica Fernandes

**Affiliations:** 1 Nuclear Medicine, Mount Sinai Hospital, New York, NY, USA; 2 Mount Sinai Hospital, New York, NY, USA

## Background

Chemotherapeutically induced myocardial injury continues to pose a major problem. The exact mechanism of drug-related cardiomyopathy (DRC) is still poorly understood. An early diagnosis of cardiotoxicity and the ability to predict patients at risk are important for preventing chronic heart failure. The guidelines for non-invasive cardiac imaging to screen for DRC show limitations in early identification of patients at risk, currently relying on left ventricular (LV) ejection fraction (EF) for establishing high risk. Contrast enhanced cardiac magnetic resonance imaging (ce-CMR) may offer a stronger screening approach, especially since it can assess the right ventricle (RV) and detect early changes in response to chemotherapy.

## Methods

51 patients (31 men, 20 women) with various types of cancer, who underwent chemotherapy with a variety of regimens, were assessed with ce-CMR within 6 months of chemotherapy. Ce-CMR results for both RV and LV were compared with published data (Hudsmith et al., JACC, 2005) using the 1-sample Student t test. A p value <0.05 was considered significant. Descriptive statistics are described as percentages.

## Results

In patients, EF was lower in both ventricles: RVEF=54.8% and LVEF= 53.6% vs. controls: RVEF=61% and LVEF= 69%, (p=0.002, p<0.001, respectively). Right and left stroke volumes (SV) were also lower: RVSV= 69.1 mL, LVSV= 76.3 mL, vs. controls: RVSV and LVSV=104 mL (p≤ 0.001). Both ventricles demonstrated decreased end diastolic volumes (EDV): RVEDV=132.8 ml, LVEDV=144.4 ml, vs. controls: RVEDV =173 ml and LVEDV=150ml, though only statistically significant in the RV (p<0.001). An elevated end systolic volume (ESV) was also noted: RVESV=70.1 mL, LVESV=67.4 mL vs. controls: RVESV=69 ml and LVESV=47 ml, though only statistically significant in the LV (p<0.001).

Interestingly, with respect to wall motion abnormalities, 4 (8%) patients demonstrated segmental abnormalities in the RV. In the LV, 15 patients (29%) demonstrated wall motion abnormalities, involving multiple segments (13/15 at the base, 13/15 at mid level, and 14/15 in the apex). Only 1 (2%) patient demonstrated delayed enhancement (DE) in the RV, while 10 patients (20%) demonstrated DE in the LV, also involving multiple segments: 10/10 in the basal LV, 8/10 in the mid LV, and 7/10 in the apex.

## Conclusions

Decreased RVEF, LVEF and ventricular volumes, as well as wall motion abnormalities and presence of DE regions in non-coronary pattern of distribution, may represent early changes secondary to chemotherapy. Earlier detection of DRC may allow for earlier initiation of treatment, and prevent progression of heart failure.

## Funding

None.

**Figure 1:**
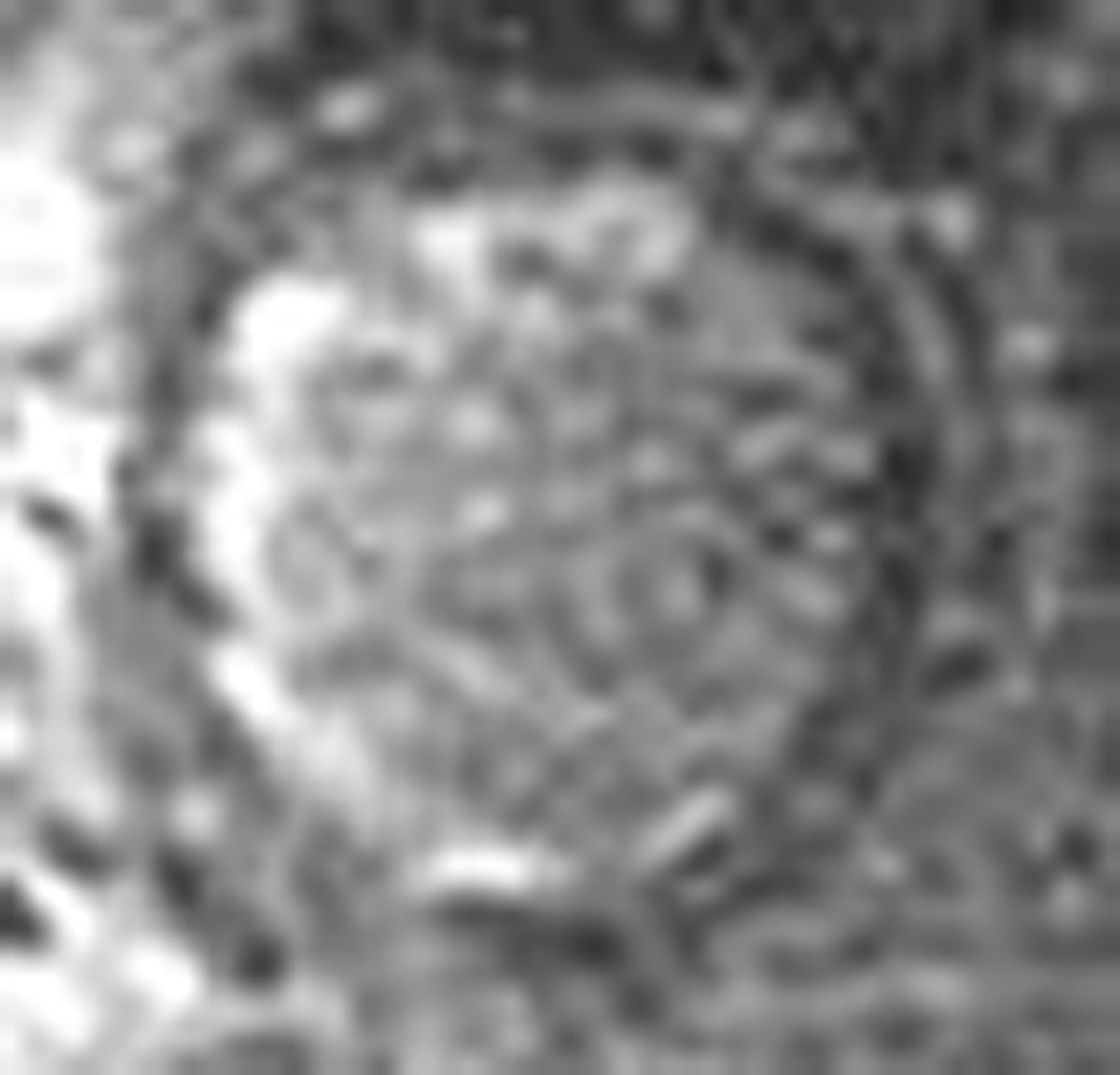
Delayed enhancement involving the left ventricle in a 61 year old male with Acute Myelogenous Leukemia.

